# Resistance Analysis of a 3-Day Monotherapy Study with Glecaprevir or Pibrentasvir in Patients with Chronic Hepatitis C Virus Genotype 1 Infection

**DOI:** 10.3390/v10090462

**Published:** 2018-08-28

**Authors:** Teresa I. Ng, Tami Pilot-Matias, Rakesh Tripathi, Gretja Schnell, Preethi Krishnan, Thomas Reisch, Jill Beyer, Tatyana Dekhtyar, Michelle Irvin, Liangjun Lu, Armen Asatryan, Andrew Campbell, Betty Yao, Sandra Lovell, Federico Mensa, Eric J. Lawitz, Jens Kort, Christine Collins

**Affiliations:** 1 AbbVie, Inc., North Chicago, IL 60064, USA; tami.pilot-matias@abbvie.com (T.P.-M.); rakesh.l.tripathi@abbvie.com (R.T.); gretja.schnell@abbvie.com (G.S.); preethi.krishnan@abbvie.com (P.K.); thomas.reisch@abbvie.com (T.R.); jill.beyer@abbvie.com (J.B.); tanya.dekhtyar@abbvie.com (T.D.); michelle.irvin@abbvie.com (M.I.); liangjun.lu@abbvie.com (L.L.); armen.asatryan@abbvie.com (A.A.); andrew.campbell@abbvie.com (A.C.); betty.yao@abbvie.com (B.Y.); sandra.lovell@abbvie.com (S.L.); federico.mensa@abbvie.com (F.M.); jens.kort@abbvie.com (J.K.); christine.collins@abbvie.com (C.C.); 2 Texas Liver Institute, University of Texas Health San Antonio, San Antonio, TX 78215, USA; lawitz@txliver.com

**Keywords:** glecaprevir, pibrentasvir, monotherapy, resistance, HCV, ABT-493, ABT-530

## Abstract

Glecaprevir (an NS3/4A protease inhibitor) and pibrentasvir (an NS5A inhibitor) are potent and pangenotypic hepatitis C virus (HCV) direct-acting antivirals. This report describes the baseline polymorphisms and treatment-emergent substitutions in NS3 or NS5A detected in samples from HCV genotype 1-infected patients receiving 3-day monotherapy of glecaprevir or pibrentasvir, respectively. None of the NS3 polymorphisms detected in the 47 baseline samples collected prior to glecaprevir monotherapy conferred reduced susceptibility to glecaprevir. The NS3 A156T substitution, which conferred resistance to glecaprevir but had low replication efficiency, emerged in one genotype 1a-infected patient among the 35 patients with available post-baseline sequence data. Baseline NS5A polymorphisms were detected in 12 of 40 patients prior to pibrentasvir monotherapy; most polymorphisms were single-position NS5A amino acid substitutions that did not confer resistance to pibrentasvir. Among the 19 patients with available post-baseline NS5A sequence data, 3 had treatment-emergent NS5A substitutions during pibrentasvir monotherapy. All treatment-emergent NS5A substitutions were linked multiple-position, almost exclusively double-position, substitutions that conferred resistance to pibrentasvir. Replicons engineered with these double-position substitutions had low replication efficiency. In conclusion, resistance-conferring substitutions emerged in a small number of genotype 1-infected patients during glecaprevir or pibrentasvir monotherapy; unlike other NS5A inhibitors, pibrentasvir did not select single-position NS5A substitutions during monotherapy.

## 1. Introduction

Hepatitis C virus (HCV) infection is a major public health problem, with a recent report estimating that approximately 70 million individuals are chronically infected with HCV worldwide [1]. There are 7 HCV genotypes, with genotype 1 being the most prevalent globally [2,3]. Depending on various risk factors, patients with chronic HCV infection can develop serious liver diseases including cirrhosis and hepatocellular carcinoma. Sustained virologic response resulting from treatment of chronic HCV infection has been shown to be associated with a significant reduction in liver disease progression, development of hepatocellular carcinoma and mortality [4,5].

A number of HCV direct acting antivirals (DAAs) have been approved since 2011. These DAAs include inhibitors of HCV NS3/4A protease, NS5A protein, and NS5B polymerase [6,7]. Current HCV treatment regimens are generally interferon-sparing, and include 2 or 3 DAAs with different mechanisms of action with and without the addition of ribavirin (RBV). Although newer DAA combinations are highly efficacious in many HCV-infected populations, a number of them are not effective for all HCV genotypes [8,9,10,11]; some of them have reduced activity against certain baseline resistance-associated polymorphisms [12,13,14,15], and DAA resistance-conferring substitutions often emerge in patients who experience virologic failure [11,16,17,18]. There have been major efforts to discover and develop next generation interferon- and RBV-sparing DAA regimens that are convenient to take (once daily oral administration), efficacious in patients infected with all major HCV genotypes, cover broad patient populations, and exhibit a high barrier to the development of DAA resistance. The fixed-dose combination regimen of glecaprevir and pibrentasvir, recently approved in Europe, the United States, Canada, Japan and many other countries for the treatment of chronic HCV infection, is one of the few regimens that has all of these features.

Glecaprevir (ABT-493, an NS3/4A protease inhibitor identified by AbbVie and Enanta) and pibrentasvir (ABT-530, an NS5A inhibitor identified by AbbVie) are next-generation HCV inhibitors with potent antiviral activity against all major HCV genotypes in vitro, with little or no loss in activity against common single-position amino acid substitutions that confer resistance to other NS3/4A protease inhibitors or NS5A inhibitors, respectively [19,20]. A Phase 2a clinical study was conducted to assess the antiviral activity, safety, tolerability, and pharmacokinetics of multiple dose levels of glecaprevir or pibrentasvir administered as monotherapy for 3 days in treatment-naïve adults with chronic HCV genotype 1 infection with or without compensated cirrhosis [21]. Monotherapy with glecaprevir or pibrentasvir was well tolerated, and resulted in mean maximal decreases of approximately 4 log_10_ IU/mL from baseline in HCV RNA levels in patients with most of the doses evaluated. The monotherapy was followed by a 12-week treatment with ombitasvir/paritaprevir/ritonavir plus dasabuvir and weight-based RBV. This report describes the prevalence of baseline polymorphisms and treatment-emergent substitutions in NS3 or NS5A in samples from patients who received 3-day monotherapy of either glecaprevir or pibrentasvir, respectively. In addition, the changes in susceptibility conferred by these baseline polymorphisms and treatment-emergent substitutions in NS3 or NS5A to glecaprevir or pibrentasvir, respectively, were determined.

## 2. Materials and Methods

### 2.1. Compounds

Glecaprevir, pibrentasvir and ombitasvir were synthesized at AbbVie. Daclatasvir, elbasvir, ledipasvir, and velpatasvir were purchased from MedChem Express (Monmouth Junction, NJ, USA).

### 2.2. Study Design

This was a Phase 2a, dose-ranging study (Study M13-595, ClinicalTrials.gov identifier NCT01995071, 26 November 2013) that investigated the antiviral activity, safety, tolerability and pharmacokinetics of glecaprevir or pibrentasvir administered as monotherapy for 3 days in HCV genotype 1-infected treatment-naïve patients with or without compensated cirrhosis [21]. The study included 2 sub-studies based on the DAA evaluated: The glecaprevir sub-study had 6 dosing arms (Arms 1, 2, 3, 4, 5 and 11), and the pibrentasvir sub-study had 5 dosing arms (Arms 6, 7, 8, 9, and 10) (Table 1). Multiple doses were evaluated in patients without cirrhosis and a single dose was evaluated in patients with compensated cirrhosis for each DAA. The 3-day monotherapy was followed by 12 weeks of combination treatment with ombitasvir/paritaprevir/ritonavir plus dasabuvir and weight-based RBV. Eighty-nine patients were enrolled in the study, with 49 patients in the glecaprevir dosing arms and 40 patients in the pibrentasvir dosing arms. Nine patients were enrolled in Arm 3 of glecaprevir monotherapy, but one patient discontinued the monotherapy early and the patient’s samples were not included in this analysis. Among the samples from the remaining 88 patients who completed the 3-day monotherapy, the samples from a patient in Arm 11 of glecaprevir monotherapy were too low in viral titer (<1000 IU/mL) to be analyzed. Therefore, samples with sufficient viral titer (i.e., ≥1000 IU/mL) from 87 patients were analyzed in this study. The study was designed by the study investigators and sponsor (AbbVie) according to Good Clinical Practice guidelines, the Declaration of Helsinki, and applicable regulations, with institutional review board approval at all study sites. All patients provided written informed consent. The sponsor conducted the data analyses. The investigators had full access to data for review and comment.

### 2.3. Viral Sequence Analysis

This report focuses on the viral sequence analysis of samples collected at baseline and during the 3-day monotherapy period. Before dosing of the study drug for each day, a plasma sample was collected from each patient on visit day 1 (baseline), day 2 (after 1 day of monotherapy), day 3 (after 2 days of monotherapy), and day 4 (after 3 days of monotherapy). Only baseline and post-baseline samples with an HCV RNA level ≥1000 IU/mL were sequenced in order to allow accurate assessment of the amplification products. Baseline sample from each patient with sufficient viral titer was sequenced. Among the post-baseline samples collected from each patient on each day during the 3-day monotherapy, the latest sample with an HCV RNA level ≥1000 IU/mL was sequenced.

Viral RNA was isolated from plasma samples, and full-length sequences of the relevant HCV targets, NS3/4A or NS5A from glecaprevir or pibrentasvir monotherapy samples, respectively, were RT-PCR amplified and analyzed by Sanger sequencing (population sequencing; detection sensitivity: ~15%) as described previously [21]. Clonal sequencing (approximately 80 clones for each sample) was performed as described previously [22,23] on selected samples to determine the prevalence and linkage of substitutions in samples with multiple amino acid substitutions. For results of clonal sequencing, amino acid substitutions detected in at least 2 clones from each sample are reported. For clones with multiple amino acid substitutions that were linked in the same HCV genome in a sample, the substitutions are denoted by “+”. Substitutions at the following amino acid positions that are associated with resistance to one or more members of the HCV NS3/4A protease or NS5A inhibitor class were included in the analysis: Substitutions at NS3 amino acid positions 36, 43 (genotype 1a only), 54, 55, 56, 80, 122, 155, 156, 168, and 170 for glecaprevir, and substitutions at NS5A amino acid positions 24, 28, 29, 30, 31, 32, 58, 62, 92, and 93 for pibrentasvir.

A polymorphism in a baseline sample was determined by comparison of the viral amino acid sequence of a baseline sample to that of the appropriate HCV subtype reference amino acid sequence (genotype 1a-H77: NC_004102; genotype 1b-Con1: AJ238799) for a given DAA target. A treatment-emergent HCV substitution is defined as a substitution that was not present at baseline and was observed at a post-baseline time point.

### 2.4. Effects of Amino Acid Substitutions on the Antiviral Activity of Different DAAs in HCV Replicon Cells

Bicistronic subgenomic HCV replicons were constructed with mutations encoding amino acid substitutions of interest in NS3 or NS5A to evaluate their susceptibility to different DAAs in transient replicon assays as previously described [19,20]. Mutagenesis was performed using the Change-IT Multiple Mutation Site Directed Mutagenesis Kit (USB, Cleveland, OH, USA) or by cloning a synthesized DNA fragment encoding the amino acid substitution(s). After mutagenesis was confirmed by sequence analysis, the plasmids were linearized and transcribed using the TranscriptAid T7 High Yield Transcription Kit (Fermentas, Waltham, MA, USA). Huh-7 derived cells [22] were transfected with the replicon RNAs; inhibition of replication of these HCV replicons by different DAAs was measured using the luciferase assay (Luciferase Assay System; Promega, Madison, WI, USA). Each 50% effective concentration (EC_50_) value and the corresponding standard deviation were calculated from results of at least 3 independent experiments, with at least 2 replicates in each experiment. The EC_50_ value was calculated using nonlinear regression curve fitting to the 4-parameter logistic equation in the Prism 4/5 software (Graph-Pad, La Jolla, CA, USA). Replication efficiency of a replicon with amino acid substitution(s) was calculated as a percentage of the replication efficiency of the respective wild-type replicon as described previously [19,20]. Fold of resistance of a replicon with amino acid substitution(s) was calculated as the ratio of its mean EC_50_ value relative to the mean EC_50_ value of the corresponding wild-type replicon as described previously [19,20].

## 3. Results

### 3.1. Patients with Available HCV Sequencing Data

This 3-day monotherapy study included 11 dosing arms, with patients in Arms 1–5 and 11 receiving different doses of glecaprevir, and those in Arms 6–10 receiving different doses of pibrentasvir (Table 1). A total of 88 patients, with 8 patients in each arm, completed the 3-day monotherapy. To determine the impact of baseline polymorphisms and treatment-emergent substitutions on the responses to monotherapy with glecaprevir or pibrentasvir in these patients, the full-length NS3/4A or NS5A sequences, respectively, from their baseline and post-baseline samples (with HCV RNA ≥1000 IU/mL) were analyzed by population sequencing. All baseline HCV samples collected could be sequenced with the exception of one sample with low viral titer (HCV RNA < 1000 IU/mL) from a genotype 1a-infected patient in Arm 11. Therefore, sequence data were available for baseline samples from 47 patients receiving glecaprevir monotherapy and 40 patients receiving pibrentasvir monotherapy (Table 1). Forty out of 47 (85%) and 33 out of 40 (83%) patients in the glecaprevir and pibrentasvir monotherapy studies, respectively, were infected with HCV genotype 1a; the rest of the patients were infected with HCV genotype 1b. This study was conducted in the United States, where genotype 1a is the most prevalent subtype of HCV genotype 1 [24]. During the monotherapy period, there were fewer patients with post-baseline samples with sufficient viral titer (HCV RNA ≥ 1000 IU/mL) for sequence analysis in each of the dosing arms for pibrentasvir than for glecaprevir (Table 1). A total of 19 out of 40 (48%) post-baseline samples from patients treated with pibrentasvir monotherapy as compared with 35 out of 47 (74%) post-baseline samples from glecaprevir monotherapy could be evaluated by population sequencing. Post-baseline sample from only 1 out of the 8 patients dosed with the highest concentration of pibrentasvir (Arm 8, 400 mg dose) had sufficient viral titer to be analyzed (Table 1).

### 3.2. Prevalence of Baseline Polymorphisms

The prevalence of each baseline polymorphism at NS3 or NS5A amino acid positions associated with resistance to inhibitors of the respective HCV inhibitor class in samples from patients in the glecaprevir or pibrentasvir monotherapy study, respectively, is summarized in Supplementary Table S1 or Table S2. The overall prevalence of baseline polymorphisms in NS3 or NS5A in samples from this study was similar to those reported for other HCV clinical studies [22,23,25,26]. In addition, the distribution of baseline NS3 or NS5A polymorphisms appeared to be similar among the different arms.

Among the 47 baseline samples from patients in the glecaprevir dosing arms, baseline NS3 polymorphisms were detected in 26 (55%) samples (Table 2 and Supplementary Table S1). Single NS3 polymorphisms were present in 18 of the 26 samples, while the remaining 8 samples each had 2 or 3 NS3 polymorphisms (Table 2). Q80K (43%) was the most prevalent genotype 1a NS3 polymorphism, whereas V170I (57%) was the most prevalent genotype 1b NS3 polymorphism. Other polymorphisms were also detected at NS3 amino acid positions 54, 55, 56, 80, 122 and 170, but not at positions 36, 43, 155, 156, and 168. There were no apparent differences in the viral load declines among patients with no, single or double NS3 baseline polymorphisms during glecaprevir monotherapy as reported by Lawitz et al. [21].

**Figure 1:**
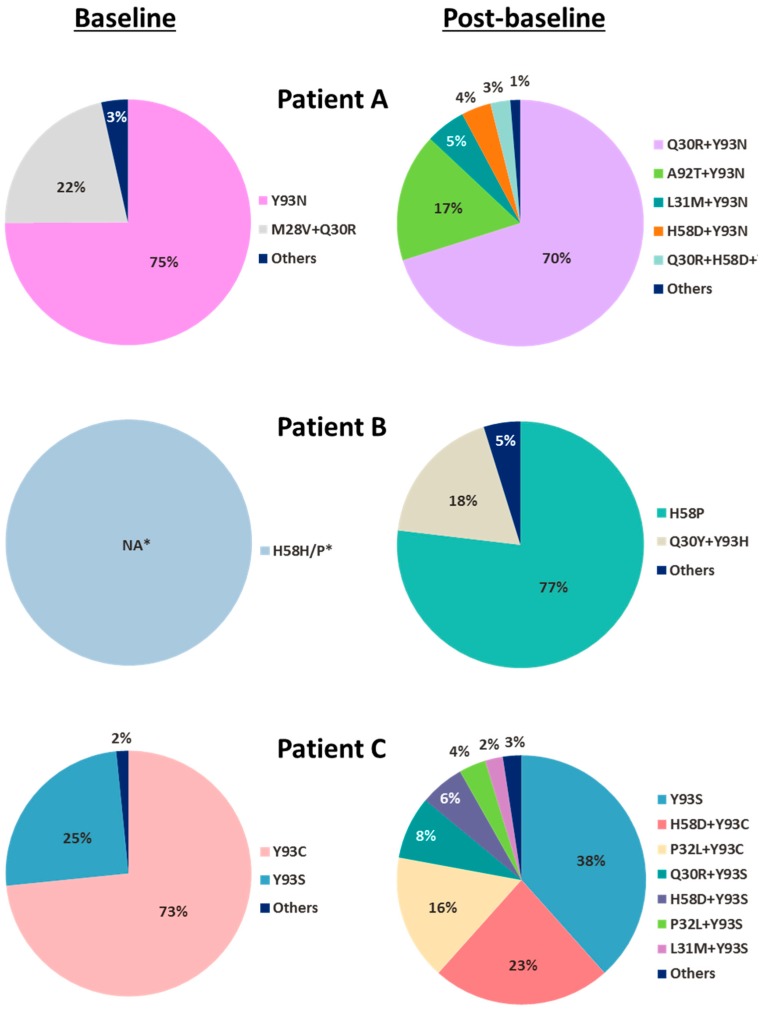
Baseline and post-baseline NS5A substitutions from 3 patients with multiple treatment-emergent NS5A substitutions during pibrentasvir monotherapy. For the 3 patients (Patients A–C) who were identified by population sequencing to have multiple treatment-emergent NS5A substitutions during pibrentasvir monotherapy, their baseline and post-baseline samples were further analyzed by clonal sequencing (approximately 80 clones for each sample) to determine the prevalence and linkage of the NS5A substitutions in each sample. Clonal sequencing was not performed on the baseline sample from Patient B as only 1 substitution (H58P) was detected by population sequencing. For clones with multiple amino acid substitutions that were linked on the same HCV genome, the substitutions are denoted by “+”. * Percentage not available (NA); clonal sequencing not done.

Among the 40 baseline samples from patients in the pibrentasvir dosing arms, NS5A polymorphisms were detected in 12 (30%) samples, with the prevalence of M28V, Q30R, L31M and Y93C/H/N/S being 5% (2/40), 5% (2/40), 2.5% (1/40), and 7.5% (3/40; 1 patient had a mixture of Y93C/S), respectively (Table 3 and Supplementary Table S2). Eight of these 12 patients had an NS5A polymorphism at a single amino acid position while 4 patients had NS5A polymorphisms at 2 or 3 amino acid positions in their baseline samples (Table 3). One genotype 1a-infected patient had M28V, Q30R, and H58P while another genotype 1a-infected patient (Patient A, Table 3 and Figure 1) harbored M28V, Q30R, and Y93N in their respective baseline samples. Two genotype 1b-infected patients also had NS5A polymorphisms at 2 amino acid positions: 1 patient had substitutions at positions 31 and 58, while the other had substitutions at positions 58 and 62 (Table 3). None of the patients had baseline polymorphisms at amino acid positions 24, 29, 32, and 92. The presence of single or multiple baseline NS5A polymorphisms had no impact on overall viral load declines in patients during pibrentasvir monotherapy as reported previously [21].

### 3.3. Treatment-Emergent Substitutions

In the glecaprevir monotherapy arms, of the 30 genotype 1a-infected and 5 genotype 1b-infected patients with available post-baseline NS3 sequence data (Table 1), 4 genotype 1a-infected patients had treatment-emergent NS3 substitutions, all of whom also had baseline polymorphisms (Table 2). One genotype 1a-infected patient in Arm 3 (700 mg dose) had a treatment-emergent substitution of A156T in the sample collected on visit day 3 prior to dosing (i.e., after 2 days of glecaprevir monotherapy) (Table 2), with a prevalence of 19% within the patient’s viral population (14/74 clones) as detected by clonal sequencing (data not shown). Treatment-emergent substitutions at amino acid positions 54 and 80 were detected in 3 other patients.

In the pibrentasvir monotherapy arms, of the 14 genotype 1a-infected and 5 genotype 1b-infected patients with available post-baseline sequence data (Table 1), 3 genotype 1a-infected patients (Patient A in 400 mg Arm 8, and Patients B and C in 120 mg Arm 10) each had multiple treatment-emergent substitutions at amino acid positions 30, 31, 32, 58, 92 and/or 93 in NS5A (Table 3). Each of the 3 patients had baseline polymorphisms in NS5A and acquired additional NS5A substitutions at resistance-associated amino acid positions during monotherapy. To determine the prevalence and linkage of NS5A substitutions in the baseline and post-baseline samples of the 3 patients who had multiple treatment-emergent substitutions in NS5A, clonal sequencing was performed. Each of the patients had different NS5A amino acid substitutions in baseline and post-baseline samples (Figure 1). Linkage of amino acid substitutions on the same HCV genome in each sample is denoted by “+”. At baseline, Patient A had the single-position substitution Y93N in 75% of the clones and the double-position substitution M28V + Q30R in 22% of the clones; Patient C had the single-position substitutions Y93C and Y93S in 73% and 25% of the clones, respectively. Clonal sequencing was not performed on the baseline sample from Patient B as only one substitution (H58P) was present. All of the treatment-emergent substitutions detected in these 3 patients were linked multiple-position NS5A substitutions: Almost all were double-position substitutions with the exception of a minor population (3%) of triple-position substitution (Q30R + H58D + Y93N) in Patient A. None of the patients had the same treatment-emergent substitutions: The predominant treatment-emergent substitutions in each patient were Q30R + Y93N (70%) and A92T + Y93N (17%) in Patient A, Q30Y + Y93H (18%) in Patient B, and H58D + Y93C (23%) and P32L + Y93C (16%) in Patient C.

### 3.4. Susceptibility of Baseline or Treatment-Emergent NS3 Substitutions to Glecaprevir

To determine if the NS3 amino acid substitutions detected in baseline or post-baseline samples from patients receiving glecaprevir monotherapy conferred reduced susceptibility to glecaprevir, HCV genotype 1a and 1b replicons engineered with these NS3 substitutions were tested against glecaprevir in vitro. The Q80K substitution in genotype 1a, which was the most prevalent baseline polymorphism (Table 2 and Supplementary Table S1), did not confer resistance to glecaprevir (Table 4). Only one treatment-emergent NS3 substitution, A156T from a genotype 1a-infected patient, conferred resistance (1361-fold) to glecaprevir. However, the replication efficiency of a genotype 1a replicon engineered with the NS3 A156T substitution was only 5.2% of that of the wild-type replicon. None of the other amino acid substitutions detected in this study, whether from baseline or post-baseline samples, reduced the susceptibility to glecaprevir in vitro. Overall, treatment-emergent resistance-conferring NS3 substitution (A156T) was detected in only 1 out of 47 patients in the glecaprevir monotherapy study, but the emergence of this substitution did not appear to impact the viral load decline in this patient at the time points monitored as reported previously [21].

### 3.5. Susceptibility of Baseline or Treatment-Emergent NS5A Substitutions to Pibrentasvir

HCV replicons engineered with NS5A substitutions detected in baseline or post-baseline samples from patients receiving pibrentasvir monotherapy were tested against pibrentasvir in vitro. All single-position substitutions in genotype 1a or 1b NS5A at amino acid position 28, 30, 31, 32, 58, 62, 92, or 93 evaluated in this study are known to be associated with resistance to a number of NS5A inhibitors [10,11,16,17,23,25,26,27,28,29,30]. Most of the HCV replicons engineered with one of these single-position NS5A substitutions had pibrentasvir EC_50_ values that were similar to (≤2-fold increase in EC_50_) those of the respective wild-type replicons, while genotype 1a replicon with Y93H or Y93N each had pibrentasvir EC_50_ value that was 7-fold of that of the wild-type replicon (Table 5).

The replication efficiency and susceptibility to pibrentasvir of genotype 1a HCV replicons with double-position treatment-emergent NS5A substitutions varied depending on the combination of substitutions (Table 5). All replicons with these double-position NS5A substitutions had lower replication efficiency in vitro than those of replicons with the corresponding single-position substitutions or no substitutions. All 8 replicons engineered with different treatment-emergent double-position NS5A substitutions had replication efficiency ≤~30% of that of the wild-type replicon, with 5 of them having replication efficiencies ≤5% of that of the wild-type replicon. For replicons engineered with treatment-emergent double-position NS5A substitutions that had sufficient replication to allow for drug susceptibility testing, their susceptibility to pibrentasvir decreased by 131- to 1969-fold (Table 5).

At baseline, Patient A had a mixture of HCV with single-position NS5A substitution Y93N (7-fold reduced susceptibility to pibrentasvir) and double-position NS5A substitution M28V + Q30R (no reduction in susceptibility to pibrentasvir) (Figure 1, Table 5). In the sample collected on visit day 4 prior to drug dosing, a mixture of multiple-position NS5A substitutions emerged in this patient with Q30R + Y93N and A92T + Y93N being the more prevalent treatment-emergent substitutions (Figure 1). Double-position NS5A substitution Q30R + Y93N conferred 131-fold resistance to pibrentasvir, while susceptibility of a replicon with A92T + Y93N substitution to pibrentasvir could not be determined due to low replication efficiency (Table 5). A slightly less robust viral RNA decline was observed in the sample collected from Patient A than in those from other patients in the same dose group (400 mg dose) on visit day 4 prior to drug dosing: ~3 log_10_ IU/mL decline for Patient A versus a mean decline of ~4 log_10_ IU/mL for the dose group [21]. This was likely due to the resistance-conferring substitutions that emerged during pibrentasvir monotherapy rather than the baseline polymorphisms, as the baseline polymorphisms of Y93N and M28V + Q30R had minimal to no impact on the susceptibility to pibrentasvir in vitro (Table 5). Although both Patient B and Patient C had treatment-emergent double-position NS5A amino acid substitutions that conferred resistance to pibrentasvir in vitro (Table 5), these substitutions appeared to have no impact on their viral load declines at the time points monitored as reported previously [21]. In summary, treatment-emergent NS5A substitutions conferring resistance to pibrentasvir were detected in 3 patients in the pibrentasvir monotherapy study, and the impact of these substitutions on the viral load declines in these 3 patients ranged from low to none.

### 3.6. Susceptibility of NS5A Amino Acid Substitutions to Pibrentasvir and Other Approved NS5A Inhibitors

In this study, treatment-emergent amino acid substitutions that were associated with resistance to glecaprevir or pibrentasvir were found to be uncommon among genotype 1-infected patients treated with the respective monotherapy. The low number of patients with resistance-conferring treatment-emergent NS3 amino acid substitutions with glecaprevir monotherapy was consistent with the improved in vitro resistance profile demonstrated by glecaprevir in comparison with other approved NS3/4A protease inhibitors, including paritaprevir and grazoprevir, against a panel of NS3 amino acid substitutions as reported previously [20]. To compare the resistance profile of pibrentasvir with those of other approved NS5A inhibitors, pibrentasvir was tested along with daclatasvir, ombitasvir, elbasvir, ledipasvir, and velpatasvir against a panel of NS5A amino acid substitutions that emerged in HCV genotype 1-infected patients treated with these other NS5A inhibitors [10,11,16,17,24,25,26,27,28,29,30]. This panel included single- as well as double-position substitutions at amino acid positions 28, 30, 31, 32, 58, 92 and/or 93 in NS5A of HCV genotype 1a or 1b (Table 6). The EC_50_ value of pibrentasvir (0.72 pM) for wild-type genotype 1a replicon was 4- to 15-fold lower than those of all of the other NS5A inhibitors tested, while that for wild-type genotype 1b replicon (1.9 pM) was 2- to 6-fold lower than those for daclatasvir, elbasvir, and velpatasvir, similar to that for ledipasvir, and 2-fold higher than that for ombitasvir. Pibrentasvir demonstrated ≤7-fold decrease in activity against all of the genotype 1a and 1b replicons with single-position NS5A substitutions tested in this study, while all of the other NS5A inhibitors demonstrated significantly lower activity than pibrentasvir against replicons with these substitutions (Table 6). For example, genotype 1a Y93H and Y93N, which are common substitutions known to emerge with HCV regimens containing other NS5A inhibitors, conferred 201- to 66,740-fold resistance to other NS5A inhibitors but ≤7-fold increase in EC_50_ to pibrentasvir (EC_50_ ≤ 5.1 pM) (Table 5 and Table 6). In addition, while genotype 1a M28T and Q30E substitutions conferred >100-fold and >1000-fold resistance, respectively, to daclatasvir, ombitasvir, and ledipasvir, they conferred lower levels of resistance (13- to 58-fold) to elbasvir and velpatasvir, and no resistance to pibrentasvir. Genotype 1a A92K substitution, which was reported to emerge in patients who experienced virologic failure with a regimen containing daclatasvir [31] or ledipasvir [32], had no impact on the activity of pibrentasvir, but conferred 7188- to 112,034-fold resistance to all of the other NS5A inhibitors tested (Table 6). The resistance levels of pibrentasvir were also significantly lower than those of the other approved NS5A inhibitors against the double-position NS5A substitutions tested in this study. Other NS5A inhibitors generally manifested >1000-fold reduction in activity to genotype 1a double-position substitutions, and >100-fold reduction in activity to genotype 1b double-position substitutions (Table 6). In contrast, pibrentasvir showed little loss in activity (≤6-fold increase in EC_50_) against 5 of the 10 genotype 1a replicons with double-position substitutions; for the other 5 genotype 1a replicons with double-position substitutions, pibrentasvir demonstrated 17- to 260-fold reduction in activity. In addition, pibrentasvir demonstrated no reduction in activity against 4 genotype 1b replicons each with different double-position NS5A substitutions. Although velpatasvir had an improved resistance profile compared with daclatasvir, ombitasvir, elbasvir, and ledipasvir, its activity against all of the NS5A amino acid substitutions tested in this study was significantly lower than that of pibrentasvir, which included the genotype 1a single-position substitutions A92K, Y93H and Y93N (409- to 54,665-fold resistant to velpatasvir versus ≤7-fold resistant to pibrentasvir), and all of the genotype 1a double-position substitutions (5.9- to 166,675-fold resistant to velpatasvir versus 0.41- to 260-fold resistant to pibrentasvir).

## 4. Discussion

In this study, resistance analysis was conducted on all baseline and post-baseline samples with sufficient viral titer from genotype 1-infected patients who received 3 days of glecaprevir or pibrentasvir monotherapy. Baseline NS3 or NS5A polymorphisms detected in these patients had no or minimal (≤7-fold reduction in activity) impact on the susceptibility of HCV to glecaprevir or pibrentasvir, respectively, in vitro. The frequency of patients with treatment-emergent resistance-conferring substitutions detected in glecaprevir or pibrentasvir monotherapy study was much lower than those in monotherapy studies with other inhibitors in the respective HCV inhibitor class [22,23,25,26,30,33,34,35,36,37]. All of the treatment-emergent resistance-conferring NS5A substitutions identified during pibrentasvir monotherapy were linked multiple-position amino acid substitutions. Replicons engineered with the resistance-conferring NS3 or NS5A substitutions that emerged in this monotherapy study had substantially lower replication efficiency relative to the wild-type HCV replicon. Glecaprevir and pibrentasvir demonstrated no loss in activity or less reduction in activity than other members of the respective inhibitor class against common resistance-conferring substitutions in NS3 and NS5A, respectively. Findings in this resistance analysis contributed to the explanation of the favorable HCV viral load declines in patients receiving glecaprevir or pibrentasvir monotherapy.

Baseline samples from 26 out of 47 patients who received glecaprevir monotherapy had NS3 polymorphisms but none of these polymorphisms conferred resistance to glecaprevir in vitro. The most prevalent baseline NS3 polymorphism detected in this study was genotype 1a Q80K, a common NS3 polymorphism that is known to be associated with reduced efficacy with regimens containing simeprevir [14], voxilaprevir [38], or other NS3/4A protease inhibitors [27]; genotype 1a Q80K does not reduce susceptibility to glecaprevir in vitro. Among the 35 patients with post-baseline sequence data available, only 1 treatment-emergent NS3 substitution that conferred resistance to glecaprevir (A156T) was detected in a genotype 1a-infected patient receiving glecaprevir monotherapy. The presence of A156T at 19% prevalence in this patient’s viral population on visit day 3 did not have an apparent impact on the patient’s viral RNA decline [21]. The NS3 resistance profile of post-baseline samples from genotype 1-infected patients receiving glecaprevir monotherapy was in agreement with the results of in vitro resistance selection study with glecaprevir in HCV genotype 1 replicon cells: The only resistance-conferring NS3 substitutions selected by glecaprevir in vitro were A156T and A156V [20]. Consistent with the rare emergence of resistance-conferring NS3 substitutions during glecaprevir monotherapy described in this report, only 1 out of 703 (0.14%) treatment-naive genotype 1-infected patients receiving the combination regimen of glecaprevir and pibrentasvir in the Phase 3 ENDURANCE-1 study experienced virologic failure: This patient was infected with genotype 1a HCV and had a treatment-emergent A156V substitution in NS3 [39].

Treatment-emergent resistance-conferring substitutions in NS3 were less commonly detected in genotype 1-infected patients treated with monotherapy of glecaprevir than other approved HCV NS3/4A protease inhibitors [22,34,35,36,37]. Approved HCV protease inhibitors, including telaprevir, boceprevir, simeprevir, asunaprevir, paritaprevir, grazoprevir and voxilaprevir, have reduced activity against genotype 1 HCV with substitutions at NS3 amino acid positions 155, 156 and/or 168 in vitro [16,22,34,35,36,37,40,41]. These substitutions also emerged in patients who were treated with each of these protease inhibitors as monotherapy, or patients experiencing virologic failure with combination HCV regimens containing one of these protease inhibitors. For example, in clinical studies with patients infected with HCV genotype 1, substitutions at amino acid positions 155 and 156 emerged in patients treated with voxilaprevir monotherapy [37] whereas substitutions at amino acid positions 155, 156 and 168 emerged in patients who experienced virologic failure with various regimens containing grazoprevir [40,41]. The most common NS3 amino acid substitutions that emerged during paritaprevir monotherapy were R155K and D168V in genotype 1a and D168V in genotype 1b [22]. A156T was the only resistance-conferring NS3 substitution found to emerge in a single patient in the glecaprevir monotherapy study. The replication efficiency of replicons engineered with substitutions at amino acid A156 was greatly impaired in vitro [20]. Therefore, the reported prevalence of A156 substitutions as baseline polymorphisms was low [20] and even if these substitutions emerged in patients treated with regimens containing a protease inhibitor, they became undetectable in as short as 3 months after the patients experienced virologic failure [41].

Twelve out of the 40 patients who received pibrentasvir monotherapy had baseline NS5A polymorphisms; 4 of these 12 patients had multiple NS5A polymorphisms. Patients with baseline NS5A polymorphisms had similar viral RNA declines as patients without any NS5A polymorphisms during pibrentasvir monotherapy with the exception of Patient A [21], whose slightly less robust viral response on visit day 4 was likely due to the presence of treatment-emergent NS5A substitutions rather than that of the baseline NS5A polymorphisms. In studies with HCV genotype 1 replicons, single-position NS5A substitutions did not generally reduce susceptibility to pibrentasvir, and only certain combinations of double-or triple-position NS5A substitutions were shown to confer resistance to pibrentasvir [19]. Therefore, it is not surprising that almost all of the patients with single or multiple NS5A baseline polymorphisms demonstrated robust viral declines with pibrentasvir monotherapy [21]. Unlike other NS5A inhibitors, which in monotherapy studies selected both single-and multiple-position NS5A substitutions that conferred resistance to the respective NS5A inhibitors [23,25,30,33], pibrentasvir did not select any single-position NS5A substitutions during monotherapy. Among the 19 patients with available post-baseline sequence data, 3 genotype 1a-infected patients, all with pre-existing substitutions in NS5A at baseline, had treatment-emergent substitutions in NS5A during pibrentasvir monotherapy. All of the treatment-emergent substitutions found in these 3 patients were linked multiple-position, almost exclusively double-position, NS5A substitutions. The impact of these treatment-emergent NS5A substitutions on viral load declines in these 3 patients receiving pibrentasvir monotherapy appeared to range from low to none at the time points monitored [21].

The frequency of patients with treatment-emergent NS5A substitutions, all multiple-position substitutions, was low among patients receiving pibrentasvir monotherapy. This could be due to (a) the high genetic barrier to the generation of each of the multiple-position NS5A substitutions, and (b) the low viral fitness of HCV with each of these multiple-position NS5A substitutions. Consistent with the pattern of the emergence of multiple-position NS5A substitutions observed during pibrentasvir monotherapy, the single genotype 1-infected patient who experienced virologic failure in the Phase 3 ENDURANCE-1 study (combination regimen of glecaprevir and pibrentasvir) as mentioned above had the linked triple-position NS5A substitution Q30R + L31M + H58D detectable at the time of virologic failure [39].

When tested against HCV replicons engineered with NS5A substitutions known to confer resistance to other members of the NS5A inhibitor class, pibrentasvir demonstrated no loss in activity or less reduction in activity than other approved NS5A inhibitors which included daclatasvir, ombitasvir, elbasvir, ledipasvir and velpatasvir. In monotherapy studies in genotype 1-infected patients, significantly fewer patients had treatment-emergent NS5A substitutions with pibrentasvir monotherapy than with other NS5A inhibitors [23,25,26,30,33]. For example, treatment-emergent NS5A substitutions at amino acids M28, Q30, L31 and/or Y93 were detected by population sequencing in the majority of available post-baseline samples from genotype 1-infected patients (*n* = 20) receiving 5-day elbasvir monotherapy [26]. In particular, treatment-emergent substitutions at amino acid Y93 were the most prevalent substitutions (present in approximately 80% of available post-baseline samples from these patients) with elbasvir monotherapy, including Y93H and Y93N in genotype 1a (201- to 605-fold resistant to elbasvir, Table 6) as well as Y93H in genotype 1b (5-fold resistant to elbasvir, Table 6) [26]. In a 3-day monotherapy study with ledipasvir, treatment-emergent NS5A substitutions were detected by population sequencing on Day 4 in 100% of genotype 1-infected patients receiving ≥3 mg of ledipasvir (*n* = 41), with the most prevalent substitutions being Q30R and L31M in genotype 1a, as well as Y93H in genotype 1b [25], each of which confers >300-fold resistance to ledipasvir (Table 6). For the 3-day monotherapy study with ombitasvir, treatment-emergent NS5A amino acid substitutions were detected by clonal sequencing in 100% of available post-baseline samples from day 3 and day 6 visits of genotype 1a-infected patients (*n* = 8) receiving different doses of ombitasvir [23]. The predominant treatment-emergent NS5A substitutions were M28T, M28V and Q30R (58- to 8965-fold resistant to ombitasvir), while Y93C and Y93H (both >1600-fold resistant to ombitasvir) were the minor substitutions [23]. In addition, in a 3-day monotherapy with velpatasvir in genotype 1-infected patients, treatment-emergent substitutions in NS5A were detected by next-generation sequencing (NGS) at 1% sensitivity cutoff in 100% (10/10) or 100% (40/40) available “Days 2–10” post-baseline samples from patients with or without baseline NS5A polymorphisms, respectively [30]. The predominant treatment-emergent genotype 1 NS5A substitutions detected in velpatasvir monotherapy were genotype 1a Y93H (≥400-fold resistant to velpatasvir, Table 6) and Y93N (≥3000-fold resistant to velpatasvir, Table 6), seen in 67% (6/9) and 56% (5/9), respectively, of Day 5 samples collected from patients without baseline NS5A polymorphisms. In the current study, after the 3-day monotherapy with pibrentasvir in genotype 1-infected patients, treatment-emergent NS5A substitutions were detected by population sequencing in only 16% (3/19) of patients with post-baseline samples that had sufficient viral titer to be sequenced. The presence of treatment-emergent NS5A substitutions, if any, in post-baseline samples from the other 21 patients receiving pibrentasvir monotherapy could not be determined due to the rapid declines of HCV viral RNA in these patients. Importantly, while NS5A Y93H in genotype 1, especially in genotype 1a, is a common treatment-emergent single-position substitution observed in patients receiving monotherapy of other NS5A inhibitors or patients who experienced virologic failure with regimens containing other NS5A inhibitors [11,16,17,23,25,26,27,28,29,30,33], the Y93H substitution did not emerge in genotype 1-infected patients receiving pibrentasvir monotherapy.

Both population sequencing and NGS with 15% sensitivity cutoff are regarded as specific and sensitive sequencing methods to identify substitutions that can impact clinical responses to HCV DAAs [29,42,43]. Monotherapy resistance analyses for NS5A inhibitors were generally done by population sequencing [25,26,33] and the analysis for velpatasvir monotherapy was done by NGS [30]. Despite the differences in study designs and/or sequence analysis methods between the monotherapy studies with pibrentasvir and the other approved NS5A inhibitors, the low number of genotype 1-infected patients with treatment-emergent NS5A substitutions for pibrentasvir detected by population sequencing in monotherapy samples is consistent with the high cure rate observed in HCV-infected patients treated with the combination regimen of glecaprevir and pibrentasvir as discussed below.

The favorable monotherapy resistance profiles of glecaprevir and pibrentasvir in genotype 1-infected patients have translated into robust clinical efficacy with the combination of glecaprevir and pibrentasvir in patients chronically infected with HCV genotypes 1–6. In Phase 2b/3 registration studies, the overall rate of sustained virologic response 12 weeks after the end of treatment (SVR12) was 98% with the combination of 300 mg glecaprevir and 120 mg pibrentasvir for 8, 12 or 16 weeks in 2256 patients infected with HCV genotypes 1–6, including patients who were treatment-naïve or treatment-experienced (pegIFN, RBV and/or sofosbuvir) with compensated cirrhosis or without cirrhosis [44]. Additional studies also showed that the combination regimen of glecaprevir and pibrentasvir was efficacious in patients with HIV-1 co-infection [39] or chronic kidney disease [45].

## Figures and Tables

**Table 1:** Number of patients evaluated in sequence and resistance analyses.

Arm ^a^	Dose	Mean Maximal Decrease in HCV RNA (Log_10_ IU/mL)	*n*/*N* (%) ^b^	
			Genotype 1a	Genotype 1b
**Glecaprevir monotherapy**				
1	100 mg	4.1	5/7	0/1
2	400 mg	4.0	4/6	2/2
3	700 mg	4.3	6/8	-
4	200 mg	4.2	6/7	0/1
5 ^c^	200 mg	3.9	4/5	3/3
11 ^d^	300 mg	3.8	5/7	-
**Pibrentasvir monotherapy**				
6	15 mg	3.4	3/6	2/2
7	120 mg	4.5	3/7	0/1
8	400 mg	4.3	1/8	-
9	40 mg	4.1	3/6	1/2
10 ^c^	120 mg	3.9	4/6	2/2

^a^ Each arm had 8 patients who completed the 3-day monotherapy. ^b^ *N* = total number of patients with available baseline sequence data, *n* = number of patients with available post-baseline sequencing data. ^c^ Arms for patients with cirrhosis. ^d^ Baseline sequence data were available from a total of 7 patients in Arm 11 as the baseline sample from 1 patient could not be evaluated due to low viral titer (<1000 IU/mL).

**Table 2:** Baseline polymorphisms and treatment-emergent substitutions in NS3 in patients treated with 3-day glecaprevir monotherapy.

HCV Subtype	Number of Patient(s) ^a^	Arm (Dose, mg)	Baseline NS3 Polymorphism ^b^	Post-Baseline	
				Treatment-Emergent NS3 Substitution ^b,c^	Visit Day ^d^
1a	1	3(700)	T54S, V55I	None	2
	1	3(700)	V55A	None	3
	1	11(300)	V55A, Q80K	None	2
	3	2(400)/3(700)/4(200)	Q80K	NA	NA
	8	Multiple	Q80K	None	Multiple
	1	4(200)	Q80K	K80Q	2
	1	1(100)	Q80K	T54S	2
	1	3(700)	Q80K	A156T	3
	1	3(700)	Q80K, S122G	K/Q80L ^e^	2
	1	11(300)	Q80K, I170V	None	2
	1	3(700)	Q80L	NA	NA
	1	1(100)	Q80L, I170V	NA	NA
	1	1(100)	I170V	None	2
1b	1	1(100)	Y56F, S122T, V170I	NA	NA
	1	2(400)	Y56F, S122T, V170I	None	3
	1	5(200)	Q80L, V170I	None	2
	1	4(200)	V170I	NA	NA

^a^ Patients are grouped by baseline polymorphism profile. Table does not list the 21 patients without baseline polymorphisms; 16 of these 21 patients had post-baseline sequence data available, and none of the 16 patients had treatment-emergent substitutions. ^b^ For substitutions in a mixture with wild-type sequences, only the substitutions are shown. Substitutions at NS3 amino acid positions 36, 43 (genotype 1a only), 54, 55, 56, 80, 122, 155, 156, 168, and 170 were included in the analysis. ^c^ Substitutions detected in the latest sample with an HCV RNA level ≥1000 IU/mL during 3-day monotherapy. ^d^ Post-baseline samples collected prior to dosing of drug on the days of visit; e.g., a “visit day 3” sample represents a sample collected after 2 days of monotherapy. ^e^ Substitution (L) emerged in this patient with baseline sample containing a mixture of wild-type (Q) and a polymorphism (K). NA: Not available due to low viral titer (<1000 IU/mL) in samples collected at all post-baseline time points. None: No treatment-emergent substitutions detected.

**Table 3:** Baseline polymorphisms and treatment-emergent substitutions in NS5A in patients treated with 3-day pibrentasvir monotherapy.

HCV Subtype	Number of Patient ^a^	Arm (Dose, mg)	Baseline NS5A Polymorphism ^b^	Post-Baseline	
				Treatment-Emergent NS5A Substitution ^b,c^	Visit Day ^d^
1a	1	8(400)	M28V, Q30R, H58P	NA	NA
	1 ^e^	8(400)	M28V, Q30R, Y93N	L31M, H58D, A92T	4
	1	7(120)	H58N/P/T	None	2
	1 ^f^	10(120)	H58P	Q30Y, Y93H	3
	1	8(400)	E62D	NA	NA
	1	10(120)	E62D	None	3
	1 ^g^	10(120)	Y93C/S	Q30R, P32L, H58D	4
1b	1	7(120)	L31M, P58S	NA	NA
	1	10(120)	P58T, Q62E	None	2
	1	6(15)	Y93H	None	4
	1	9(40)	P58S	None	4
	1	9(40)	P58T	NA	NA

^a^ Table does not list 28 patients without baseline polymorphisms; 11 of these 28 patients had post-baseline sequence data available, and none of the 11 patients had treatment-emergent substitutions. ^b^ For substitutions in a mixture with wild-type sequences, only the substitutions are shown. Substitutions at NS5A amino acid positions 24, 28, 29, 30, 31, 32, 58, 62, 92, and 93 were included in the analysis. ^c^ Substitutions detected in the latest sample with an HCV RNA level ≥1000 IU/mL during 3-day monotherapy. ^d^ Post-baseline samples collected prior to dosing of drug on the days of visit; e.g., a “visit day 3” sample represents a sample collected after 2 days of monotherapy. ^e^ Patient A. **^f^** Patient B. **^g^** Patient C. NA: Not available due to low viral titer (<1000 IU/mL) in samples collected at all post-baseline time points. None: No treatment-emergent substitutions detected.

**Table 4:** Susceptibility of baseline or treatment-emergent NS3 substitutions to glecaprevir.

HCV Subtype	NS3 Substitutions ^a^	Glecaprevir EC_50_ (Mean ± SD, nM)	Fold Change in EC_50_^b^	Replication Efficiency ^c^ (%)
1a (H77)	Wild-type	0.21 ± 0.08	-	100
	T54S	0.20 ± 0.06	1.0	6.2
	V55I	0.05 ± 0.01	0.22	81
	Q80K	0.19 ± 0.05	0.91	91
	Q80L	0.44 ± 0.33	2.1	38
	A156T	286 ± 93	1361	5.2
	I170V	0.21 ± 0.03	1.0	77
1b (Con 1)	Wild-type	0.47 ± 0.13	-	100
	Y56F	NA	NA	1.0
	Q80L	0.30 ± 0.07	0.64	123

^a^ Replicons with the following substitutions were not available for testing: V55A and S122G in genotype 1a; S122T and V170I in genotype 1b. ^b^ Fold change relative to EC_50_ for the respective wild-type replicon. ^c^ Relative to replication efficiency of the wild-type replicon of the same subtype (100%) in transient replicon assay. NA: Data not available due to low replication efficiency (≤1%) of the replicon with the substitution. SD: Standard deviation. EC_50_: 50% effective concentration.

**Table 5:** Susceptibility of baseline or treatment-emergent NS5A substitutions to pibrentasvir.

HCV Subtype	NS5A Substitutions ^a^	Pibrentasvir EC_50_ (Mean ± SD, pM)	Fold Change in EC_50_^b^	Replication Efficiency ^c^ (%)
1a (H77)	Wild-type	0.72 ± 0.45	-	100
	M28V	1.3 ± 0.86	1.8	87
	Q30R	1.2 ± 0.62	1.7	60
	Q30Y	0.55 ± 0.11	0.77	21
	L31M	0.76 ± 0.11	1.1	141
	P32L	1.2 ± 0.43	1.7	19
	H58D	0.80 ± 0.17	1.1	66
	H58P	0.46 ± 0.06	0.64	129
	E62D	0.46 ± 0.06	0.64	104
	A92T	0.28 ± 0.03	0.39	4.1
	Y93C	1.2 ± 0.57	1.7	24
	Y93H	4.8 ± 1.5	6.7	18
	Y93N	5.1 ± 2.1	7.1	35
	Y93S	1.2 ± 0.20	1.6	3.4
	M28V + Q30R ^d^	0.82 ± 0.04	1.1	17
	Q30R + Y93N	95 ± 16	131	3.6
	Q30R + Y93S	NA	NA	<0.5
	L31M + Y93N	140 ± 34	195	31
	P32L + Y93C	NA	NA	0.5
	H58D + Y93C	168 ± 32	233	13
	H58D + Y93S	1058 ± 457	1469	2.1
	H58D + Y93N	1418 ± 279	1969	21
	A92T + Y93N	NA	NA	<0.5
1b (Con 1)	Wild-type	1.9 ± 0.80	-	100
	L31M	2.9 ± 1.2	1.5	119
	P58S	2.4 ± 1.3	1.2	80
	Y93H	1.1 ± 0.27	0.60	38

^a^ Replicons with the following substitutions were not available for testing: H58N, H58T, Q30Y + Y93H, L31M + Y93S, P32L + Y93S, and Q30R + H58D + Y93N in genotype 1a; P58T and Q62E in genotype 1b. ^b^ Fold change relative to EC_50_ for the respective wild-type replicon. ^c^ Relative to replication efficiency of the wild-type replicon of the same subtype (100%) in transient replicon assay. ^d^ “M28V + Q30R” detected in Patient A at baseline. NA: Data not available due to low replication efficiency (≤1%) of the replicon with the substitutions.

**Table 6:** Susceptibility of NS5A amino acid substitutions to pibrentasvir and other approved NS5A inhibitors.

**Genotype 1a (H77)**	**Replication Efficiency ^a^ (%)**	**Daclatasvir**	**Ombitasvir**	**Elbasvir**	**Ledipasvir**	**Velpatasvir**	**Pibrentasvir**
WT EC_50_ (pM)		7.9 ± 2.0	2.7 ± 0.80	4.8 ± 1.7	11 ± 4.0	4.1 ± 1.6	0.72 ± 0.45
NS5A substitutions				Fold change in EC_50_^b^			
M28T	89	437	8965	21	108	13	2.0
M28V	87	0.95	58	1.0	0.72	0.82	1.8
Q30E	70	10,400	1326	58	2611	17	2.4
Q30H	64	154	2.8	5.8	367	4.3	1.0
Q30R	60	178	800	14	400	4.3	1.7
L31M	141	140	1.8	5.2	339	8.2	1.1
P32L	19	388	44	6.4	202	15	1.7
H58D	66	124	243	4.5	130	2.1	1.1
A92K	2.3	74,055	11,995	7188	112,034	54,665	0.85
Y93C	24	383	1675	19	811	19	1.7
Y93H	18	2324	41,383	201	2753	409	6.7
Y93N	35	8641	66,740	605	6561	3133	7.1
Y93S	3.4	2395	7790	63	2347	53	1.6
K24R + Q30R	83	1209	950	65	2757	5.9	0.41
M28T + Q30R	32	8462	3,537,179	546	2224	34	1.6
Q30H + Y93H	35	11,270	33,490	786	2778	872	17
Q30R + L31M	49	16,785	504	2842	17,537	150	3.0
Q30R + H58D	50	64,004	320,751	4066	21,207	66	126
Q30R + Y93C	6.2	5010	43,352	328	1668	117	3.8
Q30R + Y93H	21	17,018	351,081	4263	7740	6949	260
L31M + Y93C	32	32,979	1973	2813	17,564	2965	6.1
L31M + Y93H	11	21,165	ND	4266	24,840	18,323	75
L31V + Y93H	73	275,021	32,495	13,736	61,297	166,675	94
**Genotype 1b (Con 1)**	**Replication Efficiency ^a^ (%)**	**Daclatasvir**	**Ombitasvir**	**Elbasvir**	**Ledipasvir**	**Velpatasvir**	**Pibrentasvir**
WT EC_50_ (pM)		11 ± 2.0	0.79 ± 0.25	3.2 ± 1.4	1.6 ± 0.62	4.8 ± 0.77	1.9 ± 0.80
NS5A substitutions				Fold change in EC_50_^b^			
L31V	86	2.5	8.4	0.96	22	2.1	0.77
P58D	69	3.2	577	5.1	122	3.2	1.2
Y93H	38	7.3	77	7.1	345	3.0	0.60
Y93N	52	7.8	220	2.5	250	3.8	0.62
Y93S	23	0.82	12	0.66	60	0.49	0.39
L31I + Y93H	44	311	ND	176	63,257	88	1.2
L31M + Y93H	11	1166	142	626	13,940	143	0.70
L31V + Y93H	24	1259	12,328	1040	67,323	2201	0.87
P58S + Y93H	34	51	1401	25	1101	26	0.78

^a^ Relative to replication efficiency of the wild-type replicon of the same subtype (100%) in transient replicon assay. ^b^ Fold change relative to EC_50_ for the respective wild-type replicon. WT: Wild-type. ND: Not determined.

## Supplementary Materials

The following are available online at http://www.mdpi.com/1999-4915/10/9/462/s1, Table S1: Prevalence of baseline NS3 polymorphisms in patients treated with glecaprevir monotherapy, Table S2: Prevalence of baseline NS5A polymorphisms in patients treated with pibrentasvir monotherapy.
