# Supplementary material for: Resistance Analysis of a 3-Day Monotherapy Study with Glecaprevir or Pibrentasvir in Patients with Chronic Hepatitis C Virus Genotype 1 Infection
Source: Viruses. 2018 Aug 28;10(9):462. doi: 10.3390/v10090462 (PMC6163913; doi:10.3390/v10090462)
Supplement: Supplementary file 1 [file viruses-10-00462-s001.zip › viruses-337385-SI.docx]

**Supplementary Materials**

**Table S1.** Prevalence of baseline NS3 polymorphisms in patients treated with glecaprevir monotherapy.

| **NS3 Polymorphism** | | **n/N ^a^** | | | | | | | | | |
| --- | --- | --- | --- | --- | --- | --- | --- | --- | --- | --- | --- |
|  |  | **Arm 1** | | **Arm 2** | | **Arm 3** | **Arm 4** | **Arm 5** | | **Arm 11** | **Total (%)** |
| **Genotype 1a** | | |  | | |  |  | |  |  |  |
| T54S | 0/7 | | 0/6 | | 1/8 ^b^ | | 0/7 | | 0/5 | 0/7 | 1/40 (2.5) |
| V55A | 0/7 | | 0/6 | | 1/8 | | 0/7 | | 0/5 | 1/7 ^b^ | 2/40 (5.0) |
| V55I | 0/7 | | 0/6 | | 1/8 ^b^ | | 0/7 | | 0/5 | 0/7 | 1/40 (2.5) |
| Q80K | 4/7 | | 2/6 | | 3/8 ^c^ | | 5/7 | | 0/5 | 3/7 ^c^ | 17/40 (43) |
| Q80L | 1/7 ^b^ | | 0/6 | | 1/8 | | 0/7 | | 0/5 | 0/7 | 2/40 (5.0) |
| S122G | 0/7 | | 0/6 | | 1/8 ^b^ | | 0/7 | | 0/5 | 0/7 | 1/40 (2.5) |
| I170V | 2/7 ^c^ | | 0/6 | | 0/8 | | 0/7 | | 0/5 | 1/7 ^b^ | 3/40 (7.5) |
| Any ^d^ | 6/7 | | 2/6 | | 6/8 | | 5/7 | | 0/5 | 3/7 | 22/40 (55) |
| **Genotype 1b** | | |  | |  | |  | |  |  |  |
| Y56F | 1/1 ^b^ | | 1/2 ^b^ | | - | | 0/1 | | 0/3 | - | 2/7 (29) |
| Q80L | 0/1 | | 0/2 | | - | | 0/1 | | 1/3 ^b^ | - | 1/7 (14) |
| S122T | 1/1 ^b^ | | 1/2 ^b^ | | - | | 0/1 | | 0/3 | - | 2/7 (29) |
| V170I | 1/1 ^b^ | | 1/2 ^b^ | | - | | 1/1 | | 1/3 ^b^ | - | 4/7 (57) |
| Any ^d^ | 1/1 | | 1/2 | | - | | 1/1 | | 1/3 | - | 4/7 (57) |

1. N = total number of patients with available baseline sequence data; n = number of patients with the specified NS3 baseline polymorphisms. For polymorphisms in a mixture with wild-type sequences, only the polymorphisms are shown. Polymorphisms relative to the subtype-specific reference sequence at NS3 amino acid positions 36, 43 (genotype 1a only), 54, 55, 56, 80, 122, 155, 156, 168, and 170 were included in the analysis.
2. Polymorphism detected in combination with other NS3 polymorphism(s) in each patient.
3. Polymorphism detected alone or in combination with other NS3 polymorphism(s) in each patient.
4. Any NS3 baseline polymorphisms (single or multiple) in each patient.

Table S2. Prevalence of baseline NS5A polymorphisms in patients treated with pibrentasvir monotherapy

| **NS5A**  **Polymorphism** | **n/N ^a^** | | | | | |
| --- | --- | --- | --- | --- | --- | --- |
|  | **Arm 6** | **Arm 7** | **Arm 8** | **Arm 9** | **Arm 10** | **Total (%)** |
| **Genotype 1a** | |  |  |  |  |  |
| M28V | 0/6 | 0/7 | 2/8 ^b^ | 0/6 | 0/6 | 2/33 (6) |
| Q30R | 0/6 | 0/7 | 2/8 ^b^ | 0/6 | 0/6 | 2/33 (6) |
| H58N | 0/6 | 1/7 | 0/8 | 0/6 | 0/6 | 1/33 (3) |
| H58P | 0/6 | 1/7 | 1/8 ^b^ | 0/6 | 1/6 | 3/33 (9) |
| H58T | 0/6 | 1/7 | 0/8 | 0/6 | 0/6 | 1/33 (3) |
| E62D | 0/6 | 0/7 | 1/8 | 0/6 | 1/6 | 2/33 (6) |
| Y93C | 0/6 | 0/7 | 0/8 | 0/6 | 1/6 ^b^ | 1/33 (3) |
| Y93N | 0/6 | 0/7 | 1/8 ^b^ | 0/6 | 0/6 | 1/33 (3) |
| Y93S | 0/6 | 0/7 | 0/8 | 0/6 | 1/6 ^b^ | 1/33 (3) |
| Any ^c^ | 0/6 | 1/7 | 3/8 | 0/6 | 3/6 | 7/33 (21) |
| **Genotype 1b** | |  |  |  |  |  |
| L31M | 0/2 | 1/1 ^b^ | - | 0/2 | 0/2 | 1/7 (14) |
| P58S | 0/2 | 1/1 ^b^ | - | 1/2 | 0/2 | 2/7 (29) |
| P58T | 0/2 | 0/1 | - | 1/2 | 1/2 ^b^ | 2/7 (29) |
| Q62E | 0/2 | 0/1 | - | 0/2 | 1/2 ^b^ | 1/7 (14) |
| Y93H | 1/2 | 0/1 | - | 0/2 | 0/2 | 1/7 (14) |
| Any ^c^ | 1/2 | 1/1 | - | 2/2 | 1/2 | 5/7 (71) |

1. N = total number of patients with available baseline sequence data, n = number of patients with the specified NS5A baseline polymorphisms. For polymorphisms in a mixture with wild-type sequences, only the polymorphisms are shown. Polymorphisms relative to the subtype-specific reference sequence at NS5A amino acid positions 24, 28, 29, 30, 31, 32, 58, 62, 92, and 93 were included in the analysis.
2. Polymorphism detected in combination with other NS5A polymorphism(s) in each patient.
3. Any NS5A baseline polymorphisms (single or multiple) in each patient.
